# Supplementary material for: Do mothers also “manipulate” grandparental care?
Source: PeerJ. 2018 Nov 15;6:e5924. doi: 10.7717/peerj.5924 (PMC6240433; doi:10.7717/peerj.5924)
Supplement: Supplemental Information 1 [file peerj-06-5924-s001.pdf]

Questionnaire study 1

The questionnaire deals with relationships and resemblance between grandparents and grandchildren. It is only applicable to people who have both a daughter and a son, where both have own biological children (people with grandchildren from both the son and the daughter). The results will be used in a bachelor thesis at the University of Tromsø.

The questionnaire is completely anonymous and consists of 35 questions. One has the right to opt out of responding to specific questions if desired.

1. Age:

2. Sex:

☐ Female

☐ Male

3. How many children do you have?

Girls: Boys:

4. How old is the daughter who had children first?

5. How old is the son who had children first?

6. How many grandchildren have you gotten from your daughter?

Girls: Boys:

7. How many grandchildren have you gotten from your son?

Girls: Boys:

8. What sex is your first grandchild from your daughter?

9. What sex is your first grandchild from your son?

10. How old is your first grandchild from your daughter?

11. How old is your first grandchild from your son?

12. How long does it take by bus or car to get to your daughter’s child?

13. How long does it take by bus or car to get to your son’s child?

14. How much does it cost to travel to your daughter’s child?

15. How much does it cost to travel to your son’s child?

16. How often do you meet your daughter’s child?

☐ Daily

☐ Weekly

☐ Monthly

☐ Every six months

☐ Yearly

☐ Less often

17. How often do you meet your son’s child?

☐ Daily

☐ Weekly

☐ Monthly

☐ Every six months

☐ Yearly

☐ Less often

18. Do you visit your daughter’s child more often than he/she visits you?

☐ I visit more often

☐ The children visit more often

☐ We visit each other an equal amount

☐ Don’t know

19. Do you visit your son’s child more often than he/she visits you?

☐ I visit more often

☐ The children visit more often

☐ We visit each other an equal amount

☐ Don’t know

20. Does your daughter live with the father of the child?

☐ Yes

☐ No

21. Does your son live with the mother of the child?

☐ Yes

☐ No

In the next part I will compare your grandchildren from your son and daughter, and their mental and physical resemblance to you on a scale from 1 to 7, where 1 represents the least similar, and 7 represents most similar.

Physical resemblance is defined as physical features such as face shape, body shape, posture, etc. Psychical/mental similarity is defined as similarity in behavior, thinking, talents, personality, etc.

22. On a scale from 1 to 7, how much do you feel your daughter's child resembles you physically?

1 2 3 4 5 6 7

23. On a scale from 1 to 7, how much do you feel your son's child resembles you physically?

1 2 3 4 5 6 7

24. On a scale from 1 to 7, how much do you feel your daughter's child resembles you psychologically?

1 2 3 4 5 6 7

25. On a scale from 1 to 7, how much do you feel your son’s child resembles you psychologically?

1 2 3 4 5 6 7

26. Which of the two grandchildren considered in this questionnaire do you feel physically resembles you the most.

☐ Your son’s child

☐ Your daughter’s child

27. Which of the two grandchildren considered in this questionnaire do you feel psychologically resembles you the most?

☐ Your son’s child

☐ Your daughter’s child

28. Have your grandchildren’s resemblance to you ever been pointed out by someone in your family?

Yes / No

29. If yes, who pointed it out?

30. What grandchild was mentioned?

☐ Your son’s child

☐ Your daughter’s child

31. Was your grandchild described as very similar or very different to you?

32. Has your son or daughter ever said that one of your grandchildren resembles you?

☐ Both

☐ Only son

☐ Only daughter

☐ Neither

33. If both have commented that one of the grandchildren resembles you, who have most intensively and most frequently mentioned this?

☐ Son

☐ Daughter

☐ Equal

34. Has your son or daughter ever said that one of your grandchildren is unlike you?

☐ Both

☐ Only son

☐ Only daughter

☐ Neither

35. If both have said that one of the grandchildren is unlike you, who have most intensively and most frequently mentioned this?

☐ Son

☐ Daughter

☐ Equal

Thank you for taking the time to answer this questionnaire

Questionnaire study 2

All included respondents had to be a parent of a biological child or children and at least have one biological sibling of the opposite sex that also were a parent of a biological child or children.

What is your sex?

What is your age?

How many biological children do you have?

What is the sex and age of your first child?

What is the sex and age of your second child?

Continued

How many siblings do you have?

From oldest to youngest, which number are you among your siblings?

What is the sex and age of your, in age, closest sibling?

Does your closest sibling have children and, in case, how many?

What is the sex and age of your, in age, closest sibling?

Does your closest sibling have children and, in case, how many?

Continued

How often have your children contact with their maternal grandmother:

Daily or more often?

Approximately every other day?

Once or twice a week?

On a weekly basis?

On a monthly basis?

On an annual basis?

Rarer than annual?

Not at all (dead)?

Continued for maternal grandfather, paternal grandmother and paternal grandfather.

Resemblance (read to the respondent): It is well known that “resemblance” can be interpreted in many ways. Some emphasize external traits like eye-color, hair-color, way of walking, and body-shape, while others emphasize humor, interests, gestures and temper, that is, internal traits, when evaluating resemblance. These two ways of evaluation is of equal value. When you evaluate resemblance, do you mainly emphasize internal or external traits?

Four alternative answers: (i) external traits, (ii) internal traits, (iii) equal amount of emphasis on external and internal traits, and (vi) uncertain (under the interview the latter answer was not given as an alternative, but still used).

Which of your siblings do you resemble the most?

Which of your siblings do you resemble the least?

Which of your nieces, nephew do you resemble the most?

Which of your nieces, nephew do you resemble the least?

Do you resemble your child (children) more or less than your closest, in age, sibling resemble hers/his child (children)?

Do you resemble your child (children) more or less than your next closes, in age, tsibling resemble hers/his child (children)?

Continued for all siblings

Who resemble your mother the most, your child (children) or your closest, in age, sibling's child (children)?

Who resemble your mother the most, your child (children) or your next closest sibling's child (children)?

Continued for all siblings

Who resemble your father the most, your child (children) or your closest sibling's child (children)?

Who resemble your father the most, your child (children) or your next closest sibling's child (children)?

Continued for all siblings
